# Differential Patterns of the Division of Parenthood in Chinese Family: Association With Coparenting Behavior

**DOI:** 10.3389/fpsyg.2019.01608

**Published:** 2019-07-11

**Authors:** Shengqi Zou, Xinchun Wu, Chang Liu

**Affiliations:** ^1^Faculty of Psychology, Beijing Normal University, Beijing, China; ^2^Beijing Institute of Education, Beijing, China

**Keywords:** division of parenthood, father and mother involvement, coparenting behavior, Chinese family, person-centered approach

## Abstract

The current study explored the division of parenthood in Chinese families with adolescents by identifying the parental involvement patterns in the data obtained from 786 pairs of parents. Division-of-parenthood patterns were created via factor mixture modeling using self-reported three dimensions of father and mother involvement. Three differential division-of-parenthood patterns were identified: (a) *parent-cooperation pattern*, where moderate and equivalent involvement existed between mothers and fathers; (b) *mother-dominated pattern*, where mother involvement was particularly greater than father involvement; and (c) *father-dominated pattern*, where father involvement was particularly greater than mother involvement. Families were more likely to be in the mother- or the father-dominated pattern as their levels of positive coparenting behaviors increased. By contrast, as the levels of paternal conflict behavior increased, families were likely to be in the mother-dominated pattern. This study highlighted parents’ individual parenting role, the diverse division-of-parenthood patterns in the family, and the important role of coparenting behavior.

## Introduction

The division of domestic labor has been a fundamental issue for years. Consequently, the importance of the division of domestic labor for family life and family members has been well documented in various cultural contexts (Sevilla-Sanz, 2010; Kubricht et al., 2017). For example, Greenstein (2009) corroborated that the division of domestic labor greatly affected family life satisfaction in 30 countries, including those in Europe, South America, North America, and East Asia. In addition, Oshio et al. (2013) examined the division of domestic labor on marital satisfaction in China, Japan, and Korea and affirmed that the division of domestic labor predicted marital satisfaction of men and women in these East Asian countries. However, these studies primarily hypothesized that women took the main responsibilities in domestic labor, and few researchers have studied the multiple patterns of domestic labor division in the changing contemporary world (Luo and Chui, 2018). Little work has been simultaneously done to explore the influencing factors relating to the division of domestic labor. The current study was designed to explore the differential patterns of the division of domestic labor and the predicting role of coparenting.

### The Gendered Division of Domestic Labor

The gendered division of domestic labor is the dominant theoretical perspective for the understanding of the division of domestic labor, including housework or child care, which deeply rooted in cultural and economic tradition (Cooke, 2004). This perspective posits that men monopolize or specialize in productive activities, whereas women monopolize or specialize in domestic activities. It also suggests that gender is the key organizing principle of domestic labor and that women’s participation in housework and child care is higher than that of men (Pinto and Ortiz, 2018). Meanwhile, previous studies have revealed that the discrepancy between men and women in housework still exists, although men increase their time spent in unpaid domestic work (Dribe and Stanfors, 2009). For instance, Gershuny (2000) contended that the average domestic labor participation of men is approximately one-third of women’s time contribution. According to Hofferth et al. (2013), women spent two times more time on domestic work than men more than 10 years later. Pinto and Ortiz (2018) claimed that domestic labor has remained under women’s responsibility in recent years. Therefore, the time-spent discrepancy between women and men in domestic work may gradually diminish as time goes on. The domestic labor division between women and men still represents a *zero-sum game*, i.e., high housework participation of women corresponds to men’s low participation in a family (Amato et al., 2007).

### The Diversity of Domestic Labor Division

Another view has challenged the zero-sum game. This view claims that family members are intertwined and interdependent, in which women and men may have many common activities where women’s participation in domestic labor does not mean the withdrawal of men’s (Doherty et al., 1998). Moreover, the changing of the contemporary world has also narrowed the gender gap in domestic labor. First, the time use of women and men has become similar; women have increased the time spent in the paid workforce and reduced the time spent in unpaid domestic work, whereas men have increased the time spent on unpaid routine household work and reduced the time spent on paid works concurrently (Bianchi et al., 2012). Second, numerous women and men have adopted gender-equal ideals and have adapted to a less gender-specialized use of time (Dribe and Stanfors, 2009). Therefore, the gendered division of domestic labor could not have been a common view. Simultaneously, increasing families may have promoted gender equality in domestic labor division. Furthermore, in the changing contemporary world, the division of domestic labor may be characterized by diversification, which has largely been neglected in previous studies.

The neglected of the diversity of domestic labor division is primarily due to the hypothesis of the variable-centered approach. Some studies have claimed that women tend to dedicate more time in child care and housework than men (Lamb, 2000; Luo and Chui, 2018). These studies have gained this conclusion by calculating the correlation between gender and participation in domestic labor or by directly comparing the difference of the time spent in domestic labor between women and men under the assumption that the population is homogeneous, which may not be convincing (Muthén and Muthén, 2000). These studies could have concluded from the entire sample, thereby neglecting the heterogeneity between subsamples. The person-centered approach could allow us to identify the differential pattern of domestic labor division without assuming homogeneity over the samples (Lubke and Muthe′n, 2005). Therefore, the person-centered approach is ideal for exploring the diversity of the domestic labor division patterns. Latent profile model is a typical analysis method of the person-centered approach (Berlin et al., 2014). However, this method’s conditional independence assumption is often too stringent in family and family psychology studies because the members of the family are interdependent (Kenny and Garcia, 2012). Meanwhile, factor mixture modeling (FMM) allows conditional dependence parsimoniously (Morin et al., 2010), so that it acts as an effective method for solving the current problem. Hence, this study aimed to identify the differential pattern of the division of domestic labor by performing a series FMMs.

### Patterns of the Division of Parenthood

Furthermore, previous studies may have failed to identify the differential pattern of domestic labor division because these studies only assessed the time that men and women spent in housework and paid relatively little attention to child care or parenthood (Bianchi et al., 2012). Hofferth et al. (2013) assessed the time spent of married men and women on housework, such as cooking, cleaning, and laundry and concluded that gender inequality was prevalent in the division of domestic labor. Similarly, Pinto and Ortiz (2018) evaluated the timing of various types of housework and came to the same conclusion. Generally, mothers and fathers put greater meaning to parenthood than housework, and they experienced greater enjoyment in doing child-care activities than housework (Connelly and Kimmel, 2010). Hence, mothers and fathers may want to keep their own child care by controlling spouse’s child care, which is known as the gatekeeping phenomenon (Adamsons, 2010). Thus, both parents are actively involved in parenting their children, especially in the contemporary world. Some researchers have pointed out that mothers and fathers continued to experience unequal division of parenthood, in which mothers are more involved than fathers (Lamb, 2000; Yavorsky et al., 2015). However, this view has not been fully tested via empirical study. As mentioned above, the traditional breadwinner–homemaker model in parenthood may also have largely eroded because fathers have great will to be involved or have direct involvement in parenting their children (Pleck, 2010). The current study aimed to identify the differential pattern of the division of parenthood between mothers and fathers in a family. We hypothesized that, in the gendered division of parenthood, mother involvement was higher than father involvement. Moreover, in the equal division of parenthood, mothers and fathers had equal and relatively high involvement, which were two basic division of parenthood patterns in the changing contemporary world.

Research on father involvement, which refers to the father’s individual quantity of direct and indirect participation in parenting activities, has increased substantially in the last 40 years (Pleck and Masciadrelli, 2004; Planalp and Braungart-Rieker, 2016). These studies have confirmed, on the one hand, that increased father involvement has positive benefits for children, mothers, and fathers themselves (Wilson and Prior, 2011; Barker et al., 2017). On the other hand, fathers have great will to participate in child care and have shifted their level of involvement (Parke, 2000). In this sense, the unbalanced conception of the involvement of fathers and mothers has been attenuated to certain extent (Lareau, 2002). However, differences between mother and father involvement did not necessarily diminish completely because of the multidimensional nature of parental involvement (Lamb, 2000; Gaunt and Scott, 2014). For instance, one study has validated that the differences of the time fathers and mothers spent in parenting activities, such as social activities and play, were not apparent, whereas mothers’ involvement in teaching and caring was particularly higher than that of fathers’ (Yeung et al., 2001). Therefore, identifying the differential patterns of the domestic of parenthood using the dimensions of mother and father involvement via FMM may be a key factor to be considered in the current study.

The multidimensional construct of parental involvement has been widely recognized (Lamb, 2000). However, the specific parenting activities involving mothers and fathers are inexhaustible; besides, some parenting activities are gender specific. Therefore, to construct this concept, the empirical study needs an integrated and non-gender-specific framework (Pleck, 2010). Lamb (2000) distinguished three organizational components of parental involvement–engagement, accessibility, and responsibility–depending on the extent of direct interaction between the parent and the child. Engagement is a parent’s time spent in actual one-on-one interaction with the child, which refers to parents’ direct involvement. Accessibility activities imply parental accessibility to the child rather than direct involvement, but it could transform into direct involvement. Responsibility reflects the extent to which the parent takes ultimate responsibility for the child’s welfare and care, which refers to the parents’ indirect involvement. This tripartite model has substantial contributions to parental involvement research (Pleck, 2010). Moreover, researchers have recently corroborated that the dimensions of mother and father involvement are conceptually equivalent (Fagan et al., 2014). Therefore, we planned to measure mother and father involvement under the framework of this tripartite model for identifying the division of parenthood patterns.

### Parental Involvement With Adolescent Children

Adolescence is a life stage that causes profound changes in the family according to family cycle theory (Glick, 1989). This period is where children physically and psychologically individuate from their parents (Allen, 2008). Hence, the characteristic of father and mother involvement in adolescent family may be different from that in the early childhood family. Previous studies focused on fathers or mothers’ involvement in the early childhood family mainly measured their parenting time in caring and playing with their early childhood (Gaunt and Scott, 2014), indicating that the study comparing three dimensions of parental involvement in childhood and adolescence is limited. However, Hawkins and Palkovitz (1999) pointed that a father’s direct engagement with young children could be stronger when compared to adolescent children, and effective parental involvement would sometimes be characterized by the absence of direct interaction in later parental periods. On the other hand, adolescent children do not seek access to parents (Allen, 2008), but parents get ready to receive them at any moment with any needs. Said differently, parental accessibility and responsibility would be salient in adolescence. Research in China supported these assumptions and revealed that the level of parental involvement as measured by the three constructs from high to low was accessibility, responsibility, and engagement in adolescence (Wu et al., 2014), which indicated the necessity to measure parental involvement in a comprehensive way during adolescence.

### The Chinese Cultural Background

Here, we further focused on Chinese mothers and fathers with adolescent offspring because families with an infant or preschool child have generally concentrated on direct engagement (Barry et al., 2011). Hence, the latter fails to differentiate the multidimensions of mother and father involvement. China was selected for two reasons. First, with the industrialization of contemporary China and the government policy promoting female workforce, an increasing number of mothers have been participating in paid work (Qian and Sayer, 2016). By constract, fathers have been concurrently incorporating the nurturing role into their identity; hence, the division of parenthood between fathers and mothers has achieved gender equality in China to a certain extent (Wu et al., 2012). Chinese cultural tradition, which extremely emphasized the gendered division of domestic and productive activities, has a profound influence on contemporary family life (Whyte, 1978; Shek and Sun, 2014). Consequently, the interaction between traditional and modern culture tends to diversity the division of parenthood or the pattern of father/mother involvement in Chinese family. China provides a valuable opportunity to characterize the diversity of division of parenthood in the changing contemporary world. Second, the gendered division of domestic labor has changed in many Western countries (Hook, 2010; Neilson and Stanfors, 2014). Thus, multiple patterns of domestic labor division are prevalent in this changing process. Hence, as the first step of the comparative research work for identifying differential patterns of division of parenthood among various cultures, the conclusion gained from Chinese culture could act as a reference base for Western societies and other Eastern cultures. Such a conclusion could further facilitate our comprehension of the division of domestic labor.

### Coparenting Behavior as a Predictive Factor

Numerous theoretical and empirical studies have attempted to explain why the gendered division of domestic labor persists (West and Zimmerman, 1987; Hu and Scott, 2016). Multilevel analyses have verified that microlevel factors, including individual and couple-level factors, influence the division of domestic labor between women and men (Bianchi et al., 2012; Lyonette and Crompton, 2015). However, further studies must be conducted to identify the effect of family-level factors on the division of domestic labor. The current study aimed to examine the influence of the family-level factor, coparenting, on the identified patterns of the division of parenthood.

Family system theory stresses coparenting as “the family’s executive subsystem” to describe its importance to family life (Minuchin, 1974, 1985). Coparenting can be defined as the way partners relate to each other in their roles as parents so that coparenting behavior is an important measure of this concept (Altenburger et al., 2014). Moreover, coparenting behavior emphasizes the specific behavior element in the coparenting subsystem, which refers to coparents actively supporting or undermining each other’s parenting efforts and goals (Blandon et al., 2014). McHale (1997) argued that coparenting behavior encompasses four components: family integrity behavior (parents’ behaviors to promote a sense of family togetherness), reprimand behavior (one parent supports the other parent’s parenting behavior in disciplinary activities), conflict behavior (parents having overt conflict in the presence of the child), and disparaging behavior (largely covert parent-to-child communications to bring reproach or discredit upon the other parent). Thus, the measurement of coparenting behavior not only asks each parent to rate his or her behavior with respect to the other parent but also his or her behavior toward the whole family, which directly emphasizes the executive function of coparenting. More importantly, this measurement also distinguishes covert coparenting behavior (i.e., disparaging behavior) from overt coparenting behavior (i.e., conflict behavior), which would extend the previously narrow focus on supportive or cooperative behaviors. To compare the different effects of the coparenting characteristics of fathers and mothers on family life, the current study measured coparenting behavior of fathers and mothers separately.

The ecological context of coparenting reveals that this construct is an important family-level characteristic that contributes to the function of multiple family subsystems (Feinberg, 2003). Previous studies have confirmed that coparenting has a strong and steady predictive role on mother and father involvement (Morrill et al., 2010; Goldberg, 2015). Specifically, according to the spillover-crossover hypothesis of family system theory pertaining to the intrapersonal and interpersonal transfer of affect and behavior (Erel and Burman, 1995), mother and father coparenting behavior influenced not only his or her own involvement but also their spouse’s involvement in parenting their child (Liu et al., 2016). As observed, the current study is the first to combine father/mother involvement or examine the diverse division of parenthood patterns. Few studies have investigated the contribution of mother and father coparenting behavior to the division of parenthood patterns. Given the central role of coparenting in shaping family life, the aim of the current study was to examine the effect of mother and father coparenting behavior on the division of parenthood patterns.

Mother and father coparenting behavior may exert different effects on the two hypothesized division-of-parenthood patterns. The gendered division of parenthood was characterized by mothers playing dominant role in direct and indirect parenting activities; coparenting simultaneously indicated the executive function of mothers and fathers in the family (Minuchin, 1974; Feinberg, 2003). Therefore, the increased engagement of mothers to coparenting behavior may promote the mother’s dominance in the family, thereby increasing the possibility that the family falls under the gendered division of parenthood pattern in contrast to the equal pattern of division of parenthood. On the contrary, the more coparenting behavior fathers behaved, the weaker the mother’s dominance in the family will be. Hence, the possibility that the family falls under the equal pattern of division of parenthood is higher in comparison with the gendered division of parenthood pattern.

### The Current Study and Hypotheses

This study aimed to identify differential division-of-parenthood patterns using the person-centered approach in Chinese families with adolescents. It also aimed to examine the possible effects of father and mother coparenting behavior on division-of-parenthood patterns. To reduce the impact of gender on measuring mother and father involvement, we used the comprehensive and non-gender-specific tripartite model developed by Lamb (2000) to measure father/mother involvement (Fagan et al., 2014). To compare the different roles of mothers and fathers in the coparenting subsystem, we used the construct of coparenting by McHale’s (1997) to measure the coparenting behavior of mothers and fathers to their spouse and family. Child gender, child age, number of children, and parent working hours per week were incorporated as covariates in the statistical analysis given their potential confounding effect on father/mother involvement and coparenting behavior according to the results of theoretical and empirical studies (Doherty et al., 1998; Feinberg, 2003; McClain and Brown, 2017). We hypothesized:

H1: The gendered division of parenthood and the equal division of parenthood between mothers and fathers were two basic division-of-parenthood patterns in the changing contemporary China.
H2a: The possibility that families fall under the gendered division of parenthood pattern increased as mother engaged in further coparenting behavior.
H2b: The possibility that families fall under the equal pattern of division of parenthood increased as father engaged in further coparenting behavior.

## Materials and Methods

### Participants

A total of 786 pairs of parents of adolescent children were recruited. Our sampling strategy targeted married parents with at least one adolescent child. As Table 1 shows, our sample of mothers was, on average, 40.71 years old (*SD* = 4.15), whereas the father was 43.02 years old (*SD* = 4.14). Their children had an average age of 13.65 years (*SD* = 2.50), and 47.5% of them were boys. Moreover, fathers worked an average of 48.09 h/week (*SD* = 18.13), whereas mothers worked an average of 44.09 h/week (*SD* = 18.82). A total of 71.1% of fathers had been educated to the 9th grade or above, whereas 62.5% of mothers had reached such a level of education. Of the families involved, 55.5, 35.0, and 3.6% had one, two, and three children or more, respectively. Among fathers, 65.0% had a monthly income between 2000 and 6000 RMB, and 68.3% of mothers had a monthly income of between 1000 and 5000 RMB. Furthermore, the participants’ mean subjective socioeconomic status (SSS) was 5.46 (*SD* = 1.19) for fathers and 5.49 (*SD* = 1.12) for mothers in a 10-point scale ranging from 1 (very bad) to 10 (very good) was used as measure.

**TABLE 1:** Characteristics of study participants (*N* = 786).

	**Father**	**Mother**
	***M*(*SD*)/*N* (%)**	***M*(*SD*)/*N* (%)**
**Age**	43.02(4.14)	40.71(4.75)
**Working hours per week**	48.09 (18.13)	44.09 (18.82)
**SSS (rank: 1∼10)**	5.46 (1.19)	5.49 (1.12)
**Number of children**		
One		436 (55.5%)
Two		275 (35.0%)
Three or more		28 (3.6%)
Missing		47 (5.9%)
**Monthly income**		
Under 1000 RMB	25 (3.2%)	47 (6.0%)
1000∼2000 RMB	70 (8.9%)	136 (17.3%)
2000∼3000 RMB	144 (18.3%)	205 (26.1%)
3000∼4000 RMB	161(20.5%)	121 (15.4%)
4000∼5000 RMB	114 (14.5%)	75 (9.5%)
5000∼6000 RMB	92 (11.7%)	72 (9.2%)
6000∼7000 RMB	42 (5.3%)	32 (4.1%)
7000∼7999 RMB	46 (5.9%)	14 (1.8%)
8000∼8999 RMB	16 (2.0%)	2 (0.3%)
9000∼9999 RMB	17 (2.2%)	6 (0.8%)
At least 10000 RMB	42 (5.3%)	10 (1.3%)
Missing	17 (2.2%)	66 (8.2%)
**Highest completed education**		
Primary school or lower	13 (1.7%)	36 (4.6%)
Junior high school	191 (24.3%)	235 (29.9%)
Senior high school	302 (38.4%)	268 (34.1%)
Some college or higher	249 (31.7%)	224 (28.5%)
Missing	31 (3.9%)	23 (2.9%)

### Procedure

The current study applied the strategy of convenient sampling comprising a group of fathers and mothers of adolescent students from primary and secondary school. The participating parents were recruited under the support of their children’s school. Families whose adolescent children attended school on the date of the survey were recruited to participate. Inclusion criteria were nuclear families with a child aged 10–18 years. Divorced families and single-parent families were excluded from this study. The children brought home a package of questionnaires from school. Parents were asked to provide demographic information about their family and themselves initially, and then they were asked to complete the measures assessing parent involvement and coparenting behavior. Upon completion, the child returned the questionnaires to school. Furthermore, trained psychological graduate students examined the returned questionnaires and removed participants who missed pages and responded regularly in the survey. A total of 807 families participated in the study, and 786 pairs of valid samples were retained with the 97.40% effective rate. The participating parents were free to withdraw from the research any time, and all participants gave written informed consent. This study was approved by the Research Ethics Committee of the Beijing Normal University.

### Measures

#### Parental Involvement

Mother and father involvement were measured using the inventory of parent involvement, which was based on the construction of Lamb’s theory (Wu et al., 2015). This inventory has been used widely in China (Zou et al., 2016; Liu et al., 2016), and it has also been used for measuring mother involvement because of the claim that mother and father involvement are conceptually equivalent (Fagan et al., 2014; Hou et al., 2018). The inventory included three dimensions with 56 items. Each item was described by first-person pronouns (e.g., engagement: I discuss with the child the difficulties he/she encountered in study; accessibility: I actively ask things about the child when he or she is not with me; responsibility: I help the child develop his or her own strengths.). The responses were provided on a Likert scale ranging from 0 (never) to 4 (always). Moreover, the item scores were averaged; a higher score indicated a higher level of involvement by mother or father. In this study, Cronbach’s alphas on engagement, accessibility, and responsibility were 0.93, 0.84, and 0.94 for fathers and 0.92, 0.83, and 0.93 for mothers, respectively.

#### Coparenting Behavior

The Chinese version of the Coparenting Scale was used to measure coparenting behavior (Liu et al., 2017). This scale was the revised version of the Coparenting Scale of McHale (1997). The revised version of the scale had 29 items divided into four subscales: family integrity subscale (e.g., “Make an affirming or complimentary remark about your partner to this child,” 7 items), consistent subscale (e.g., “Take a “back seat” while your partner deals with your child’s negative behavior,” 10 items), conflict subscale (e.g., “Argue with your partner,” 6 items), and disparagement subscale (e.g., “Say something clearly negative or disparaging about your partner to your child,” 6 items). The parents responded using a seven-point scale ranging from 1 (absolutely never) to 7 (almost constantly) to assess their own coparenting behavior with their spouses, and a higher score reflected a higher level of positive or negative coparenting behavior. The revised edition had good reliability and validity index (Liu et al., 2017). In this study, Cronbach’s alphas of the subscales ranged from 0.89 to 0.94 for fathers and from 0.89 to 0.93 for mothers.

### Analysis Plan

The number of missing values at the item level was lower than 10% in the collected data. We used expectation maximization (EM) as an imputation method. This method has been confirmed to work quite effectively in processing missing data (Hair et al., 1998). Subsequent data analysis was based on the imputed dataset.

Descriptive statistics and correlations were computed using SPSS 21.0, and factor mixture modeling was used to identify the patterns of the division of parenthood in the adolescent family. Models with 1 to 5 latent profiles were specified and performed using Mplus 7.11. Moreover, several fit statistics have been used to determine the optimal number of patterns: lower Akaïke Information Criterion (AIC) value, lower Bayesian Information Criterion (BIC) value, lower Adjusted Bayesian Information Criterion (ABIC) value, high entropy, significant Lo–Mendell–Rubin likelihood Test (LRT), and Bootstrap Likelihood Ratio Test (BLRT; Nylund et al., 2007; Jung and Wickrama, 2008). Finally, multinomial logistic regressions were conducted using SPSS 21.0 to test if parent coparenting behaviors would significantly predict membership in the different pattern groups after controlling for child gender, child age, number of children, and parent working hours per week.

## Results

### Preliminary Analysis

Table 2 presents the descriptive statistics (means and standard deviations) of father and mother involvement and the correlations of study variables. On average, the levels of father/mother involvement in engagement, accessibility, and responsibility were higher than the median value. The scores of mother involvement in engagement, accessibility, and responsibility were higher than the those of father involvement: [*F*_engagement_(1,785) = 130.32, *p* < 0.001; η*_*p*_*^2^ = 0.14, *F*_accessibility_ (1,785) = 82.94, *p* < 0.001, η*_*p*_*^2^ = 0.10; *F*_responsibility_ (1,785) = 67.71, *p* < 0.001, η*_*p*_*^2^ = 0.08]. Father and mother family integrity behavior and consistent behavior were positively correlated with father/mother involvement in three dimensions from low to moderate level (*r* ranged from 0.28 to 0.65). Father and mother conflict and disparaging behaviors were negatively related to father/mother involvement across the three dimensions with low magnitude (*r* ranged from −0.11 and −0.25). Child age was negatively correlated with father and mother engagement and responsibility. The number of children in the family was negatively correlated with the three dimensions of father/mother involvement. Mother’s working hours per week were negatively correlated with mother engagement and responsibility, whereas father’s working hours per week and child gender were not correlated with parental involvement. The kurtosis and skewness values for all the variables were lower than 3 (Li et al., 2017), indicating the normal distribution of these variables. The multi-collinearity was identified by the values of variance inflation factor (VIF). The results revealed that all VIF values were less than 4.5 of all the predictor variables, which indicated that collinearity was not an issue in this study.

**TABLE 2:** Descriptive statistics of parental involvement and correlations among study variables.

**Variables**	**Fathers**			**Mothers**			**Kurtosis**	**Skewness**
				
	**Engagement**	**Accessibility**	**Responsibility**	**Engagement**	**Accessibility**	**Responsibility**		
Child gender	–0.03	0.04	0.02	0.02	0.05	–0.00	–	–
Child age	–0.18^∗∗∗^	–0.02	–0.13^∗∗∗^	–0.27^∗∗∗^	–0.06	–0.19^∗∗∗^	–1.27	0.33
Number of children	–0.16^∗∗∗^	–0.12^∗∗∗^	–0.16^∗∗∗^	–0.20^∗∗∗^	–0.14^∗∗∗^	–0.21^∗∗∗^	–	–
F_ working hour per week	–0.05	–0.07	–0.07	–0.01	–0.03	–0.01	1.86	0.11
M_ working hour per week	–0.01	–0.06	–0.04	−0.09^*^	–0.06	−0.09^*^	1.12	–0.27
F_ family integrity behavior	0.65^∗∗∗^	0.55^∗∗∗^	0.62^∗∗∗^	0.34^∗∗∗^	0.28^∗∗∗^	0.35^∗∗∗^	–0.50	–0.12
F_ consistent behavior	0.53^∗∗∗^	0.52^∗∗∗^	0.63^∗∗∗^	0.31^∗∗∗^	0.29^∗∗∗^	0.34^∗∗∗^	0.01	–0.51
F_ conflict behavior	–0.18^∗∗∗^	–0.17^∗∗∗^	–0.25^∗∗∗^	–0.10^∗∗∗^	–0.11^∗∗∗^	–0.12^∗∗∗^	0.23	0.12
F_ disparaging behavior	–0.16^∗∗∗^	–0.15^∗∗∗^	–0.20^∗∗∗^	–0.12^∗∗∗^	–0.10^∗∗∗^	–0.13^∗∗∗^	0.67	0.23
M_ family integrity behavior	–0.34^∗∗∗^	0.28^∗∗∗^	0.35^∗∗∗^	0.64^∗∗∗^	0.54^∗∗∗^	0.63^∗∗∗^	–0.38	–0.24
M_ consistent behavior	–0.32^∗∗∗^	0.31^∗∗∗^	0.36^∗∗∗^	0.49^∗∗∗^	0.46^∗∗∗^	0.57^∗∗∗^	–0.01	–0.46
M_ conflict behavior	–0.12^∗∗∗^	–0.11^∗∗∗^	–0.16^∗∗∗^	–0.13^∗∗∗^	–0.13^∗∗∗^	–0.16^∗∗∗^	0.25	0.13
M_ disparaging behavior	–0.17^∗∗∗^	–0.15^∗∗∗^	–0.19^∗∗∗^	–0.14^∗∗∗^	–0.16^∗∗∗^	–0.18^∗∗∗^	0.241	0.18
*M* (*SD*)	2.37(0.64)	2.80(0.65)	2.60(0.61)	2.65(0.57)	3.04(0.60)	2.79(0.54)	–	–
kurtosis	0.37	0.35	0.82	0.54	0.59	1.14	–	–
skewness	–0.29	–0.59	–0.53	–0.37	–0.64	–0.59	–	–

F, father; M, mother. ^*^p < 0.05, ^∗∗^*p* < 0.01, ^∗∗∗^*p* < 0.001.

### Patterns of Parental Involvement

According to the fit indices of FMM, including AIC, BIC, ABIC and entropy (Table 3), the three-class solution was the best fit model in the current study. As LMR and BLRT tend to overestimate the number of classes (Nylund et al., 2007), the current study has not taken these fit indices into consideration. In addition, the average latent class probabilities of this solution ranged from 0.92 to 0.94. Thus, group membership was well differentiated. Finally, after considering the intuitive and substantive nature of the classes, we chose the three-class solution as the best fitting model.

**TABLE 3:** Fit indices from factor mixture modeling.

	**One-class**	**Two-class**	**Three-class**	**Four-class**	**Five-class**
AIC	11359.15	10613.55	10229.91	10032.43	9926.83
BIC	11443.75	10730.23	10379.25	10212.45	10141.51
ABIC	11385.99	10650.84	10277.63	10090.60	9995.44
Entropy	Na	0.820	0.876	0.821	0.843
LMR(*p*)	Na	< 0.001	0.13	0.04	0.03
BLRT(*p*)	Na	< 0.001	< 0.001	< 0.001	< 0.001

**FIGURE 1:**
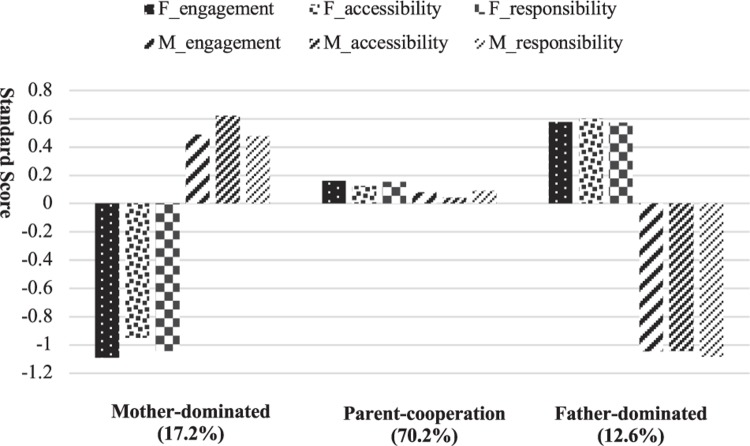
Patterns of Parental Involvement in Chinese Adolescent Family. F, father; M, mother.

Figure 1 illustrates the level of father/mother engagement, accessibility, and responsibility of the three-class solution. Most families (70.2%) were classified in the parent-cooperation pattern, which showed that mother and father had equal and moderate levels of involvement. In the second pattern (17.2%), mothers reported a high level of involvement, whereas fathers reported a low level of involvement. First, the parents with a high level of involvement had more shared time and responsibility with their children than those with a low level of involvement. Second, these parents were the primary caregivers of the children. Thus, making decisions related to the child is convenient. We named this pattern the mother-dominated pattern. The third pattern (12.6%) named the father-dominated pattern indicated that fathers had a high level of involvement, whereas mothers had a low level of involvement.

### Predictors of Patterns

Table 4 exhibits the multinomial logistic regression results. The parent-cooperation pattern was designated as the reference group in the data analysis. Fathers who had a high level of family integrity behavior were more likely to be in the father-dominated pattern and less likely to be in the mother-dominated pattern in comparison with fathers who had a low level of family integrity behavior. As the levels of mother family integrity behavior increased, families were more likely to be in the mother-dominated pattern and less likely to be in the father-dominated pattern than in the parent-cooperation pattern. Similarly, father consistent behavior had the same effect on the patterns of parental involvement with father family integrity behavior, and mother consistent behavior had no effect on the possibility of mother-dominated patterns in comparison with the parent-cooperation pattern. Contrary to positive coparenting behavior, families were more likely to be in the mother-dominated pattern than the parent-cooperation pattern as the levels of father conflict behavior increased. However, mother conflict behavior had no effect on the patterns of parental involvement. Finally, mother and father disparagement behavior did not significantly distinguish the parent-cooperation pattern from the other two patterns.

**TABLE 4:** Multinomial logistic regression of assessing predictors of patterns of parental involvement.

	**Mother-dominated Pattern**			**Father-dominated Pattern**		
		
**Predictor**	***b*(*SE*)**	**OR**	**95%CI**	***b*(*SE*)**	**OR**	**95%CI**
Child gender(girl)	−0.15⁢(0.26)	0.86	0.51∼1.45	−0.02⁢(0.29)	0.98	0.55∼1.72
Child age	−0.02⁢(0.05)	0.98	0.89∼1.09	0.01 (0.06)	1.01	0.90∼1.13
Number of children	−0.43⁢(0.24)	0.65	0.40∼1.04	−0.06⁢(0.25)	0.94	0.58∼1.54
Father’s working hours per week	0.01 (0.01)	1.01	0.99∼1.02	−0.01⁢(0.01)	0.99	0.97∼1.01
Mother’s working hours per week	−0.01⁢(0.01)	0.99	0.98∼1.01	0.01 (0.01)	1.01	0.99∼1.03
Father family integrity behavior	−1.09 (0.17)***	0.34	0.24∼0.47	0.49 (0.19)*	1.63	1.12∼2.38
Father consistent behavior	−0.37 (0.15)*	0.69	0.52∼0.93	0.38 (0.19)*	1.46	1.01∼2.12
Father conflict behavior	0.39 (0.19)*	1.47	1.02∼2.13	−0.12⁢(0.24)	0.89	0.56∼1.41
Father disparagement behavior	−0.29⁢(0.20)	0.75	0.51∼1.11	0.20 (0.24)	1.22	0.77∼1.94
Mother family integrity behavior	0.95 (0.19)***	2.59	1.79∼3.74	−0.79 (0.18)***	0.45	0.32∼0.65
Mother consistent behavior	0.07 (0.17)	1.07	0.77∼1.48	−0.38 (0.17)*	0.68	0.49∼0.95
Mother conflict behavior	−0.09⁢(0.17)	0.91	0.65∼1.27	0.33 (0.21)	1.39	0.91∼2.10
Mother disparagement behavior	0.33 (0.26)	1.39	0.96∼2.01	−0.44⁢(0.0.23)	0.64	0.41∼1.02
Cox and Snell *R*^2^	0.30					
Nagelkerke *R*^2^	0.38					

The reference category is Parent-cooperation Pattern. ^*^p < 0.05, ^∗∗^*p* < 0.01, ^∗∗∗^*p* < 0.001.

## Discussion

Understanding the division of domestic labor in parenthood is essential in family study (Yavorsky et al., 2015). The current study identified three meaningful differential patterns of the division of parenthood in Chinese family with adolescents: parent-cooperation, mother-dominated, and father-dominated patterns. Moreover, the current study revealed that the positive coparenting behavior of one parent could facilitate their own dominance in parenting their children. Only fathers’ conflicting behavior toward mothers would deprive the former of dominance in parenting activities. Our results suggested that the division of parenthood had a family difference, which updated the conventional wisdom that mothers are the primarily caregivers of children. Finally, the current study revealed that as the characteristic of the executive subsystem, coparenting behavior had an important role in the family.

### Division of Parenthood Patterns

From the point of the variable-centered approach, the current study corroborated that mothers are generally more involved in engagement, accessibility, and responsibility than fathers. This finding again supported the gendered division of domestic labor (Cooke, 2004; Pinto and Ortiz, 2018) and further indicated that the division of domestic labor in the field of child care and parenthood was also female dominated (Bianchi et al., 2012). By employing a person-centered approach (Lubke and Muthe′n, 2005), the current study identified three differential patterns of the division of parenthood between mothers and fathers, which showed the diversification of the division of parenthood in the changing contemporary China.

Most families, surprisingly, were characterized by the parent-cooperation pattern, wherein mothers and fathers had moderate and equivalent levels of involvement in parenting their adolescent children. This finding was reflective of the advancement of parenthood’s cultural conceptualizations in the contemporary world (Pleck, 2010). From the mid-1970s, the new nurturing father actively involved in the daily care of their children replaced the breadwinner father solely focusing on economic responsibility (Lamb, 2000). The gender roles of mothers have simultaneously expanded to embrace economic responsibility (Yavorsky et al., 2015). In this sense, mothers and fathers have exhibited equal social or family roles, and they have been coparents of their children. However, this finding was also reflective of the policy context in China, where the government explicitly implemented gender equality policy (Qian and Sayer, 2016). Hence, mothers and fathers had equal involvement in parenting their children. In addition, the current study asserted that each parent only performed moderate instead of high involvement in parenting their children partly because mothers and fathers were their children’s coparents, and they shared child-care responsibilities by cooperating with each other.

The mother-dominated pattern was characterized by low father involvement and high mother involvement. This pattern was reflective of the global traditional gender hypothesis that men take care of things outside the family, whereas women take care of things inside the family (Shek and Sun, 2014). However, the features of this pattern supported the claim of the maternal gatekeeping perspective wherein mothers are the primary caregivers of the child, and they possess privilege and more domestic power than fathers (Allen and Hawkins, 1999; Puhlman and Pasley, 2013). Furthermore, mothers may inhibit increased father involvement in parenting to maintain their central position in the family (Makusha and Richter, 2016). This pattern was also consistent with our general comprehension of the division of domestic labor in parenthood. However, the proportion of this pattern (17.2%) was much lower than expected. It suggested that women’s ideology about fathers’ role has changed to a certain extent. For instance, an increasing number of mothers perceived that fathers were important to their children’s development and had good parenting abilities. Hence, they have facilitated fathers’ active participation in parenting their children (Fagan and Barnett, 2003; Altenburger et al., 2018).

The two patterns mentioned above were consistent with our hypothesis of the division of parenthood. However, the father-dominated pattern was not part of our hypothesis, which corresponded to the mother-dominated pattern. This pattern was characterized by a high level of father involvement and a low level of mother involvement. Thus, fathers and mothers were the primary and secondary parents, respectively. This pattern mostly resembled a special family type called stay-at-home father families, in which fathers take primary responsibility for household and child care (Kramer and Kramer, 2016). The parenting nature of the father-dominated pattern was equivalent to the features of the stay-at-home family in parenting to a certain extent. However, most fathers in the current study had full-time jobs and worked an average of 48.09 h/week (*SD* = 18.13). The existence of this pattern may be partly because adolescent children just need a low-level of day-to-day caring and a high level of responsibility for adolescent welfare that fathers are willing to take (Pleck, 2010). However, this possibility must be further studied in the families with younger children. The proportion of this pattern was small (12.6%). However, the new nurturing father model had been recognized by some contemporary parents. Furthermore, this pattern supported the assumption that the gatekeeping perspective of parenting should be extended to include the concept of paternal gatekeeping (Adamsons, 2010).

### The Predicting Role of Coparenting Behaviors

Under the framework of family system theory (Minuchin, 1974, 1985), we explored the possible effects of parent coparenting behavior to patterns of parental involvement. We corroborated that the positive coparenting behaviors of fathers and mothers, including family integrity and consistent behaviors, could promote the parent’s own dominance in parenting their children compared to the spouse from a family level perspective. Coparenting is the characteristic of the executive subsystem, which underlines the significance of coparenting to family life (Minuchin, 1974; Feinberg, 2003). Previous studies have affirmed that positive coparenting promoted positive parent–child interactions and increased parental individual involvement in parenting activities (Liu et al., 2016; Pilkington et al., 2019). These studies have emphasized coparenting as a family-level factor for influencing individual involvement, but less is known about the dynamics of the individual behaviors of fathers and mothers during the coparenting process. Although the coparenting behaviors of fathers and mothers were likely to mutually influence each other (McHale and Lindahl, 2011), the current study further indicated that parents’ individual positive behaviors within coparenting relationship affected the family pattern of the division of parenthood between fathers and mothers, which emphasized effect of the individual behavior of fathers and mothers in the coparenting subsystem on the family-level parenting division patterns.

As a dimension of positive coparenting behavior, the consistent behavior of fathers and mothers had a different effect on the division of parenthood patterns. Specifically, the consistent behavior of fathers increased the possibility that the family falls under the father-dominated pattern instead of the parent-cooperation pattern. However, mothers’ consistent behavior did not affect the possibility of mother-dominated pattern. Such contribution from fathers would be regarded as the result of maternal gatekeeping when viewed from the maternal gatekeeping hypothesis. According to this premise, mothers perform the role of the gatekeeper who is the primary decision-maker in the child care and household, and mothers are more influential than fathers in structuring or controlling family processes (Allen and Hawkins, 1999; Puhlman and Pasley, 2013). Simultaneously, researchers have confirmed that mothers may contribute more to the interaction of competitive coparenting than fathers. However, fathers may contribute more than mothers to the interaction of cooperative coparenting (Murphy et al., 2017). Fathers’ consistent coparenting behavior means that fathers are supportive of the behavior and parenting decision of mothers (McHale, 1997; Liu et al., 2016). Therefore, the dominance of fathers in family work would be regarded as obedience to the decisions of mothers. In line with the proposed maternal gatekeeping hypothesis, fathers proactively follow the behavior and parenting attitudes of mothers. Mothers may then empower and push fathers to do additional childcare and housework activities, which clarify how fathers’ consistent coparenting behavior with mothers would facilitate dominant involvement in parenting. However, this hypothesis had completely neglected fathers’ roles (Doherty et al., 2000; Walker and McGraw, 2000). The consistent coparenting behavior of fathers may basically be the result of fathers’ careful choice (Zou et al., 2016). Thus, fathers agree with the parenting decision of mothers rather than obeying mothers unconditionally. These two possibilities suggested further exploration of the mechanism of the relationship between coparenting behavior and division of parenthood patterns.

Finally, we affirmed that families were more likely to fall under the mother-dominated pattern as father conflict behavior increased. Conflict coparenting behavior reflects overt interparental disagreement and conflict (McHale, 1997). Empirical and theoretical studies have revealed that coparenting conflict decreased the levels of positive parenting behavior (Fagan and Palkovitz, 2011; Fagan and Cabrera, 2012). This finding was consistent with our result, which illustrated that father conflict coparenting behavior was related to decreased father involvement. However, our results also confirmed that father conflict behavior was related to increased mother involvement. The current study has extended our general recognition of the efficacy of coparenting conflict. Upon exploring the coparenting conflict by fathers and mothers from the perspective of the behavioral dimension, we validated that the coparenting conflict behavior of one parent may be related to the positive parenting behavior of the other parent, which supported the crossover hypothesis of family system theory that affect and behavior can be transferred from person to person in a certain system (Pedro et al., 2012). Only father conflict behavior affected the patterns of parental involvement. This finding further supported the maternal gatekeeping hypothesis that fathers were in a secondary position and that mothers were the primary decision-makers in child care and had more family power. Father conflict coparenting behavior refers to fathers as behavioral agents who take the initiative to quarrel with mothers (McHale, 1997). Given that mothers are the primary decision-makers in the family, they may withdraw their empowerment and take care of the child solely if fathers frequently disagree and conflict with them in terms of caring and parenting. Moreover, father and mother disparagement behavior did not significantly distinguish the parent-cooperation pattern from the other two patterns. One possible answer to this question was that disparagement behavior was covert and not easily realized (Liu et al., 2016).

### Limitations and Implications

This study had considerable strength, including the investigation of parenting behavior in families with adolescents, the application of the person-centered approach, the conceptualization of parental involvement as multidimensional, and the use of the behavioral dimensions of coparenting by fathers and mothers. However, it had some limitations that must be acknowledged. First, the current study was a cross-sectional study. On the one hand, the measurement at a single time-point cannot be used to explore how the latent profiles of parental involvement may shift over time. On the other hand, the causal relationship between coparenting behavior and the patterns of division of parenthood cannot be inferred. For instance, we cannot rule out the possibility that the mother’s dominant position in parenthood leads to a high level of father conflict coparenting behavior. Second, the parents completed self-reported questionnaires only. This measure was easily influenced by social desirability, the internal reference framework or stereotype of the responders. Hence, fathers may overestimate their level of involvement and claim the largest proportion of parent-cooperation pattern. Future studies could use multiple methods and informants to measure parental involvement. Third, all participated families in the current study were recruited from China. Therefore, the findings cannot be directly applied to Europe, America, and other countries. Cross-cultural research is needed to further comprehend the similarities and differences in the division of domestic labor and parenthood. Finally, the current study explored the relationships between coparenting behaviors and division of parenthood patterns, which neglected the mechanism between them. The control variables of this study may moderate the relationships. For instance, the sex-matching effect model claims that fathers and mothers affect the development of same-sex children stronger than that of opposite-sex children (Li and Meier, 2017), suggesting the possibility that the same effect in the parenthood division may exist. Moreover, relationship quality or family variables (such as martial satisfaction) may mediate the relationships (Liu and Wu, 2018), benefiting in explaining the reasons for the relationships.

Despite these limitations, the current study made us understand the division of parenthood in the contemporary China and the interaction within the family. The three-differential pattern of mother and father involvement in the current study showed the existence of variations in the division of parenthood at a family level. It updated our traditional cognition of the division of parenthood that women solely took the responsibilities of parenting their children and further indicated that the division of parenthood patterns has been changing from only one pattern to divers in the contemporary China. This study also highlighted that fathers and mothers were coparents in many Chinese families and that the new nurturing role of fathers has been recognized by contemporary parents. In addition, the findings also have implications for Western countries. Increased families have been becoming more accepting of working mothers and egalitarian gender roles in Western counters, such as the United States (Donnelly et al., 2016), which is consistent with the findings that the majority of families in China have been characterized as parent-cooperation patterns. Moreover, Western society and Chinese had gender equality at the macro-level and the differences in family life between them gradually decreased (Yu and Lee, 2013; Ho et al., 2018). Because of these similarities, the current study suggests that the intervention programs focusing on coparenting may be an effective method for promoting equality of domestic labor division in Western and Chinese families.

## Data Availability

The datasets generated for this study are available on request to the corresponding author.

## Ethics Statement

This study was carried out in accordance with the recommendations of the Ethics Committee of the Beijing Normal University with written informed consent from all subjects. All subjects gave written informed consent in accordance with the Declaration of Helsinki. The protocol was approved by the Ethics Committee of the Beijing Normal University.

## Author Contributions

SZ was involved in the conceptualization, data collection, data analysis, interpretation, and writing. XW was involved in the conceptualization, ethical approval, and interpretation. CL was involved in the data analysis and writing.

## Conflict of Interest Statement

The authors declare that the research was conducted in the absence of any commercial or financial relationships that could be construed as a potential conflict of interest.
